# A Leaf Selfie: Using a Smartphone to Quantify Leaf Vulnerability to Hydraulic Dysfunction

**DOI:** 10.3390/plants9020234

**Published:** 2020-02-11

**Authors:** Francesco Petruzzellis, Martina Tomasella, Andrea Miotto, Sara Natale, Patrizia Trifilò, Andrea Nardini

**Affiliations:** 1 Dipartimento di Scienze della Vita, Università di Trieste, Via L. Giorgieri 10, 34127 Trieste, Italy; fpetruzzellis@units.it (F.P.); martina.tomasella@units.it (M.T.); ANDREA.MIOTTO@studenti.units.it (A.M.); SARA.NATALE@phd.units.it (S.N.); 2 Dipartimento di Scienze chimiche, biologiche, farmaceutiche e ambientali, Università di Messina, Viale Ferdinando Stagno d’Alcontres 31, 98166 Messina, Italy; ptrifilo@unime.it

**Keywords:** leaf, drought, embolism, vein, vulnerability, smartphone, optical method

## Abstract

Accurate predictions of species distribution under current and future climate conditions require modeling efforts based on clear mechanistic relationships between climate variables and plant physiological functions. Vulnerability of leaves to xylem embolism is a key mechanistic trait that might be included in these modeling efforts. Here, we propose a simple set-up to measure leaf vulnerability to embolism on the basis of the optical method using a smartphone, a light source, and a notebook. Our data show that this proposed set-up can adequately quantify the vulnerability to xylem embolism of leaf major veins in *Populus nigra* and *Ostrya carpinifolia*, producing values consistent with those obtained in temperate tree species with other methods, allowing virtually any laboratory to quantify species-specific drought tolerance on the basis of a sound mechanistic trait.

## 1. Introduction

Functional traits are useful tools for investigating plant–environment relationships [1,2], with the aim of explaining species-specific distribution ranges under current and future climate conditions, and selecting genotypes better adapted to specific climatic and edaphic situations [3,4,5]. Functional traits, defined as morphological, physiological, or phenological characteristics, can be measured at the individual level and at different scales, and from the cell to the whole-organism [6,7]. Leaf-level traits, frequently included in ecological studies, range from leaf lifespan to leaf nutrient concentration, with specific leaf area (SLA) probably representing the single most commonly measured plant trait globally [8].

A shortcoming of functional traits is that they often do not correlate in a straightforward way to a specific plant function. SLA merges information on carbon costs for leaf construction and light capture [9], nutrient availability and use [10], drought resistance [11], and protection against herbivores [12]. Hence, SLA should be not considered as a ‘trait’, but rather as a ‘syndrome’, subtending correlations and trade-offs emerging from different leaf functions including water transport, photosynthesis, and defense [13]. Accurate predictions of species distribution as a function of current climate and projected climate changes require clear mechanistic relationships between climate variables, functional traits, and physiological functions [14,15]. In this sense, it is proposed that ecological disciplines should progressively move from functional traits to the adoption of ‘mechanistic’ traits, i.e., plant features whose function can be clearly physiologically defined, operating at broad phylogenetic and ecological spatial and temporal scales [16].

Mechanistic traits are seldom included in ecological studies focused on large species’ assemblages, probably due to the time-consuming and sometimes expensive nature of procedures required to measure them. A good example is provided by relationships between species distribution and water use strategies. Species distribution and vegetation composition are shaped and constrained by water availability [17,18], and the correlation between water availability and species occurrence/performance is driven by mechanistic traits related to water use and drought tolerance [19,20]. Traits related to species-specific drought tolerance are of particular interest for studies addressing the impact of climate change on plant distribution in areas, such as the Mediterranean, where climate projections portend increasing risks of severe and repeated drought events [21]. In particular, two physiological traits have been reported to closely correlate with plant tolerance to drought, namely the leaf turgor loss point and the vulnerability to xylem embolism [22,23].

Turgor loss point (TLP) indicates the critical water potential value inducing turgor loss in plant cells [24,25]. Plants with lower TLP values generally thrive better in arid environments compared to species with higher TLP values [26] and have a higher survival probability under extreme drought episodes [27]. TLP is derived from leaf water potential isotherms (also known as pressure–volume curves) [24], a time-consuming procedure allowing for measurements of only a relatively low number of replicates. More recently, psychrometer-based estimates of leaf osmotic potential (π), followed by calculations based on known general regression models between π and TLP [28], or on corrections based on leaf dry matter content [29], have allowed for fast and reliable estimates of TLP. These new approaches are supporting the introduction of this parameter in ecological studies involving large numbers of species or samples at different spatial scales [30,31,32].

The above example illustrates how methodological and conceptual advances in techniques used to measure important mechanistic traits can promote the introduction of these traits into large-scale ecological studies. Besides TLP, one of the most relevant physiological features subtending plant adaptation to habitats with contrasting water availability is the vulnerability of the xylem system to embolism formation [33]. Long-distance water transport in plants is based on negative pressures generated by transpiration and transmitted along continuous water columns in the xylem. Water under tension is metastable, and an air phase (embolism) can be pulled in a functioning conduit from a nearby gas-filled compartment through inter-vessel pit membranes [34]. The likelihood of embolism formation increases at decreasing xylem pressure, i.e., under conditions of high transpiration and low soil water availability [35]. Hence, resistance to embolism is a key adaptation for plants thriving in warm and arid habitats [36]. Resistance to xylem embolism comes at significant carbon costs for the plant, correlated to the production of more numerous but narrower conduits, with thick cell walls to avoid implosion under high tension [37]. It is thus not surprising that evolution has generally selected against high resistance to xylem embolism in plants occupying humid habitats [38]. This trade-off [11,39] provides a powerful tool to predict species-specific occurrence and dominance in areas characterized by different water availabilities, on the basis of species-specific resistance to xylem embolism formation [22,40,41].

Quantification of species-specific vulnerability to xylem embolism is commonly based on measurements of ‘vulnerability curves’ (VCs), i.e., plots of organ (stem, root, leaf) hydraulic conductance versus water potential [42]. On the basis of VCs, the value of xylem pressure inducing 50% loss of hydraulic conductance (Ψ_50_) can be calculated and used as a reliable indicator of species’ tolerance to hydraulic dysfunction [33]. Embolism quantification is generally made via destructive hydraulic techniques [43], which are time consuming and potentially prone to artefacts [44]. In fact, in recent years, hydraulic measurements have been frequently coupled to in vivo observations of embolism formation using micro-computed X-ray tomography (microCT) [45,46,47]. While microCT provides accurate estimates of critical xylem pressures triggering embolism formation, related costs and difficulties in accessing facilities strongly limit the applicability of this technique to large species’ assemblages. More recently, a new low-cost optical method has been developed that allows for observation and quantification of embolism formation in leaf veins in a non-destructive way [48]. The method is based on the detection of changes in light transmittance through xylem conduits upon the transition from the water-filled to the gas-filled status, and has been shown to produce estimates of Ψ_50_ consistent with those obtained with more expensive and labor-intensive procedures [48]. The optical method requires sequential observations of the leaf xylem network upon progressive dehydration and this is generally achieved using a light-transmission microscope, a scanner, or a dedicated set-up. (Further details describing the optical method and related facilities are available at http://www.opensourceov.org). Considering the potential of the optical method as a tool for rapid determination of Ψ_50_ in several species, and the related possibility of including this important mechanistic trait in ecological studies, we propose a new set-up based on a smartphone, a light-emitting diode (LED) source, and a pressure chamber. This set-up will potentially allow any laboratory to reliably measure and quantify P_50_ in leaves of diverse species on the basis of the optical method.

## 2. Results

**Figure 1:**
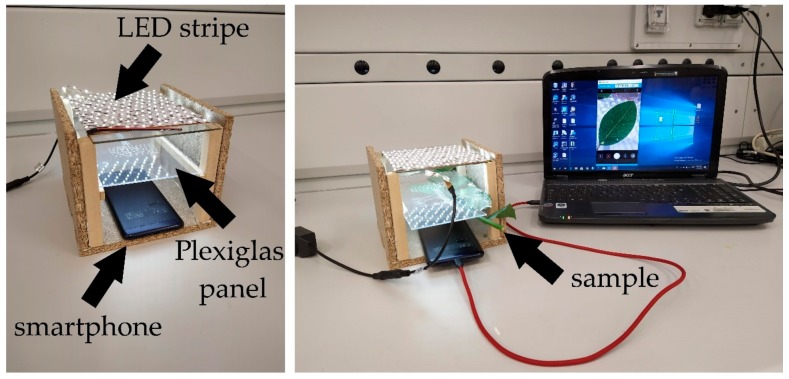
Experimental set-up. For clarity’s sake, only a small twig is shown in the photograph, but experiments were performed on branches longer than 1 m in order to avoid open-vessel artifacts (see Materials and Methods for details).

**Figure 2:**
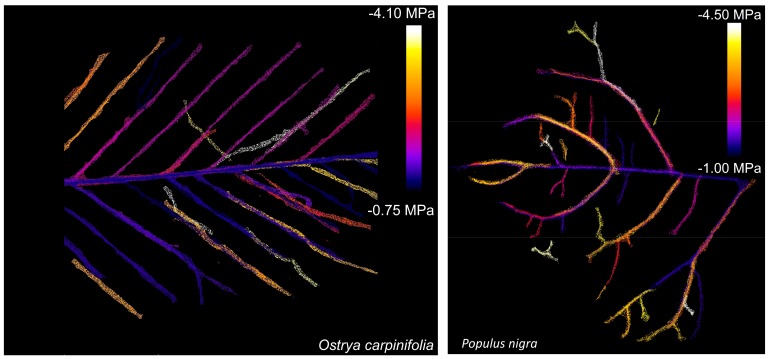
Patterns of embolism formation in the major vein network of *Ostrya carpinifolia* (**left**) and *Populus nigra* (**right**). Different colors indicate the different values of leaf water potential at which embolism was detected in the different veins.

The experimental set-up used in this study (Figure 1) allowed us to obtain clear images of the leaf vein network independently of the specific smartphone used. Veins up to the fourth order could be clearly recognized in both *Populus nigra* (*Pn*) and *Ostrya carpinifolia* (*Oc*), although embolism events were detectable only for veins up to the third order (Figure 2).

Under laboratory conditions, leaves were dehydrated over different time intervals to reach a leaf water potential (Ψ_leaf_) of about −4 MPa (see Materials and Methods). During dehydration, the first embolism events in the veins were detected at −1.0 MPa. These initial events occurred at the level of major veins, and, in particular, in the midrib. At progressively lower Ψ_leaf_, xylem embolism propagated in higher-order veins. At Ψ_leaf_ = −4.0 MPa, the major vein network appeared to be extensively embolized (Figure 2).

**Figure 3:**
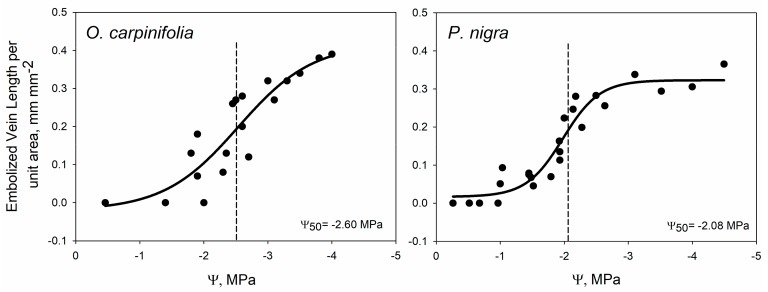
Vulnerability curves showing the embolized vein length per unit area as a function of leaf water potential, as measured in *Ostrya carpinifolia* (**left**) and *Populus nigra* (**right**). Values of leaf water potential inducing 50% embolism (Ψ_50_) are also reported (dashed line and insert in the figure).

Plotting the embolized leaf vein length per unit area (VLA_embolized_) of each leaf versus the corresponding Ψ_leaf_ produced vulnerability curves (VCs) that were clearly sigmoidal for both study species (Figure 3). At the end of the dehydration, VLA_embolized_ was about 0.3 and 0.4 mm mm^−2^ for *Pn* and *Oc*, respectively. For *Oc*, these values corresponded to the major vein density (VLA_maj_) measured after leaf clearance (data not shown). On the basis of VCs, the value of leaf water potential inducing embolism over 50% of the total length of the vein network (Ψ_50_) could be calculated. Ψ_50_ was −2.1 and −2.6 MPa in *Pn* and *Oc*, respectively.

## 3. Discussion

Our experiments showed that it is possible to measure the vulnerability to xylem embolism of the leaf vein network using a common smartphone (irrespective of its operating system), with a camera, a LED source, and a notebook. The only specific and relatively expensive piece of equipment required is the pressure chamber used to measure Ψ_leaf_ at different dehydration levels. A recent study indicates that it is possible to generate a complete optical vulnerability curve for leaves by injecting gas at known pressures into the vein network through the petiole [49]. This finding suggests that vulnerability curves might be potentially generated using any relatively cheap source of high-pressure gas to induce embolism in the vein xylem, and detecting it using the set-up shown in Figure 1. Hence, our experiments show that the optical method can be potentially used by any laboratory even when specific or expensive equipment is not available. Considering the importance of leaf vulnerability to xylem embolism as a mechanistic trait subtending species-specific resistance to drought, we feel that this set-up might encourage a larger number of laboratories to include estimates of leaf Ψ_50_ in ecological studies addressing the possible responses of plants to ongoing climate changes.

Our set-up allowed for visualization of embolism events in veins up to the third order. Changes in light transmission in minor veins could not be detected with the two smartphones used in this study. The agreement between the maximum recorded VLA_embolized_ and VLA_maj_ provides confidence on the capacity of the set-up to detect embolism in all major-order veins. While it is possible that higher-resolution cameras or different image processing would allow for the visualization of embolism events also at the minor vein level, we note that, contrary to previous conclusions based on injection of dies in the vein network [50,51,52], recent studies reveal that leaf vein embolism occurs initially in the midrib and lower-order veins [48,50], while minor veins apparently embolize only at severe stress levels. Moreover, the blockage of major veins generally has severe impacts on leaf hydraulic conductance, potentially leading to complete failure of leaf water transport capacity [53,54]. Finally, embolism events in the leaf veins occur only in proximity to, or below, water potential values inducing stomatal closure and/or turgor loss [50,55]. On the basis of these considerations, it is clear that the appearance of embolism in the major vein network already indicates a critical leaf water status, and hence relative Ψ_50_ values can be used to quantify species-specific risks of mortality under severe drought events [56].

The values of Ψ_50_ derived for *Pn* (−2.08 MPa) and *Oc* (−2.60 MPa) are in the range of those previously reported for other temperate species [22,50]. The stem Ψ_50_ for the two study species is available in the literature, and values of −2.95 MPa and −4.31 MPa have been reported for *Pn* [57] and *Oc* [58], respectively. Hence, our data collected at leaf level are consistent with those reported for stems in the sense that both indicate higher vulnerability to xylem embolism for *Pn* compared to *Oc*. This finding is in agreement with the general ecology and distribution of the study species. In fact, *Pn* is a tree adapted to grow with relatively high water availability and often in proximity to freshwater bodies. On the contrary, *Oc* is a termophylous species that can cope with seasonal water limitations, and is in fact quite common and widespread in karstic habitats characterized by edaphic aridity. This confirms that leaf Ψ_50_ values obtained with the proposed set-up can capture important information on the species-specific adaptation to contrasting water availabilities.

The optical Ψ_50_ obtained in this study is also in agreement with the available values of turgor loss point for the two species, which average −2.3 MPa in *Oc* [59] and −2.1 MPa for *Pn*. Again, this is consistent with the known relationship between leaf Ψ_50_ and turgor loss point [22], and raises confidence in the validity of Ψ_50_ values recorded in the study species.

Interestingly, in both species, leaf Ψ_50_ values turned out to be less negative than previously reported stem Ψ_50_ values. This would suggest that leaves of both *Pn* and *Oc* are more vulnerable than stems to drought-induced hydraulic dysfunction. Such a partitioning of Ψ_50_ values in different plant organs, frequently referred to as ‘vulnerability segmentation’ [60], is postulated to represent a key adaptation to prevent catastrophic hydraulic failure under severe drought conditions [61]. In fact, higher vulnerability of distal plant organs would confine embolism build-up to the periphery of the water transport system, eventually favoring stomatal closure and/or leaf shedding that, in turn, would strongly decrease transpiration rates and prevent an excessive xylem pressure drop at the stem level. Although some recent studies challenge this view [62,63], most reports confirm the existence of vulnerability segmentation [64,65,66], and our data are in agreement with these findings.

## 4. Materials and Methods

### 4.1. Plant Material and Sample Preparation

Leaf optical vulnerability (OV) curves were measured on two species, i.e., *Populus nigra* (*Pn*) and *Ostrya carpinifolia* (*Oc*). For both *Pn* and *Oc*, several sun-exposed two-year-old branches (one for each point in the OV curve, see Figure 3), were sampled from adult trees growing in the Botanical Gardens of the University of Trieste (*Oc*) and the University of Messina (*Pn*). For each species, we detached stems far longer (about 1 m) than the maximum vessel length of the two study species, thus avoiding experimental artefacts related to spurious embolism formation in open vessels. Vessel length was preliminarily assessed via the air-injection method [58], and turned out to be 5 cm for *Oc* and 20 cm for *Pn*. The cut sections were immediately put in water and additional cuts were made under water to remove any eventual embolism induced by the initial cuts [67]. Stems were moved to the laboratory and were rehydrated overnight. On the day of measurements, the water potential was measured using a Scholander pressure chamber (1505D, PMS Instrument Company, Albany, USA) on one leaf for each stem, to check that samples were fully hydrated before starting measurements.

The experimental set-up for OV curves measurement is exemplified in Figure 1 (but note that, for clarity’s sake, only a small twig is shown in the photograph, while experiments were performed on large branches). One fully expanded and well-hydrated leaf, without any damage symptoms, was selected from each stem/plant. Each leaf, still attached to the stem (approximately 60 cm), was tightly attached with transparent tape to a Plexiglas panel (Figure 1, left panel) with the abaxial surface facing the portion of the panel pierced with several small holes. This limited the sample’s movement and shrinking during dehydration and, at the same time, allowed for leaf-to-air gas exchange through the small holes in the Plexiglas panel. The Plexiglas panel with the attached leaf was placed in a custom-made box (Figure 1, left panel), with the abaxial surface of the leaf facing upwards. On the bottom of the box, we placed a smartphone (models used: Nokia Lumia 1320 and Asus Zenfone 4 Max) with the back camera facing the leaf (Figure 1, left panel). The Nokia Lumia 1320 was equipped with a 5-megapixel camera, with a resolution of 2592 × 1936 pixels and an aperture size of f2.4 f-stops. The Asus Zenfone 4 Max had a 13-megapixel camera, with a resolution of 4160 × 3120 pixels and an aperture size of f2 f-stops. An LED strip (1200 lumen) (Figure 1, left panel) was placed on the top of the custom-made box. Samples were then left to dehydrate in the laboratory for a minimum of 30 min and a maximum of 24 h. During leaf dehydration, several images were captured at increasing time intervals and then processed according to the procedure described in http://www.opensourceov.org. In this way, it was possible to capture embolism events by recording rapid changes in light transmission through the venation network [48]. Different leaves were dehydrated for different time intervals, and the water potential of the scanned leaves (Ψ_leaf_) was measured at the end of the dehydration time. Specifically, each scanned leaf was gently detached from the Plexiglas panel (Figure 1, right panel), wrapped in cling film, and separated from the branch by cutting the top of the petiole. The leaf (still wrapped in cling film) was then inserted in the Scholander pressure chamber to measure Ψ_leaf_. In this way, it was possible to couple the cumulative embolism in the leaf veins with the leaf water potential. This procedure was aimed at avoiding possible errors derived from water potential estimates on adjacent leaves that might differ from that of the observed leaf if substantial disequilibria existed across the dehydrating branch/plant.

In total, 19 and 24 leaves were scanned for *Oc* and *Pn,* respectively.

### 4.2. Image Capture and Analysis

Images were captured using transmitted light every 5 min (Windows Phone OS for the Nokia smartphone) and every 2 min (Android OS for the Asus smartphone) during sample dehydration. The smartphone was connected to a personal computer (PC) and the screen was mirrored using ProjectMyScreenApp (v 1.2, downloadable from the Microsoft website free of charge) for Windows OS, and the app Vysor (v 2.2.2, downloadable from www.vysor.io free of charge) for Android OS. (For Android smartphones, it is mandatory to make Developer Options available by tapping several times on the Build number in the Options menu of the smartphone, and then activating universal serial bus (USB) Debugging to mirror the screen on a PC. In this way, it was possible to access the smartphone’s camera directly from the PC.) From the PC, the focus and white balances were manually set in order to standardize camera settings for all the pictures captured during the experiment. Using AutoIT software (v 3.3.14.3, downloadable from https://www.autoitscript.com/site free of charge), the mouse was set to automatically click on the ‘take pictures’ command of the camera. This allowed us to take several pictures automatically at fixed time intervals with the same settings (focus and white balances). At the end of the experiment, a stack of images was obtained for each sample. Each sequence of images was then processed following the procedures described in http://www.opensourceov.org.

The aim of the processing phase was to identify changes in light transmitted through the scanned leaf veins, which corresponded to the entry point of air in the xylem conduits [48], by means of the image subtraction method available in the “OSOV Toolbox” plugin for ImageJ, which is available at http://www.opensourceov.org. In this way, we obtained a new image sequence, comprised of subtracted images, where it was possible to threshold all of the embolism events. We then measured the cumulative length of embolized veins of each image sequence to calculate the embolized vein length per unit area (VLA):

VLA_embolized_ = Cumulative length of embolized veins/Leaf area. (1)

We thus obtained couples of VLA_embolized_ and the associated water potential values for each scanned leaf, which were then used to generate OV curves. Ψ_50_ values of each species were calculated using the “fitplc” package for R software.

It should be noted that the VLA-based metric used in this study to quantify the extent of leaf embolism and build the leaf vulnerability curve differs from the most commonly used area-based metric, where embolism is quantified in terms of the percentage of leaf area showing embolized pixels. We preferred to use a VLA-based metric because the most important leaf physiological functions, such as gas exchange and photosynthetic capacity, are known to be limited by VLA, and not by the leaf area occupied by veins [68]. Hence, the calculation of VLA_embolized_ appears to be more meaningful from a functional perspective.

### 4.3. Measurements of Leaf Vein Length Per Unit Area

To compare the values of VLA_embolized_ to the total VLA, leaves of *Oc* were treated in 1 M NaOH solution for 48–72 h before a portion of leaf was cut from the central portion and cleared in 1% NaClO for 5 min. Samples were then dehydrated in a sequence of ethanol solutions at increasing concentrations (25%, 50%, 75%, and 100%), immersed in an alcoholic solution of toluidine blue (3%) overnight, and then treated in a series of ethanol solutions at decreasing concentration before microscopic slides were prepared. Images of leaf portions of ~5 mm^2^ were captured with a scanner and an optical microscope at 4× magnification equipped with a digital camera, and the VLA of major (VLA_maj_) and minor veins (VLA_min_) was measured using PhenoVein software [69].

## 5. Conclusions

Our data show how a simple and cheap set-up based on a smartphone and an LED source allows to quantify the vulnerability to xylem embolism of the leaf veins in two woody species. We are confident that the system might work equally well for different leaf types, thus making it possible for any laboratory to determine the drought tolerance of different species on the basis of a sound mechanistic trait such as Ψ_50_. We hope this will further promote the use of the optical method, and the inclusion of Ψ_50_ in ecological analyses aimed at modeling species distribution under current and future climate conditions.
